# Fat and Carbohydrate Intake over Three Generations Modify Growth, Metabolism and Cardiovascular Phenotype in Female Mice in an Age-Related Manner

**DOI:** 10.1371/journal.pone.0134664

**Published:** 2015-08-12

**Authors:** Samuel P. Hoile, Leonie M. Grenfell, Mark A. Hanson, Karen A. Lillycrop, Graham C. Burdge

**Affiliations:** 1 Academic Unit of Human Development and Health, Faculty of Medicine, University of Southampton, Southampton, United Kingdom; 2 Centre for Biological Sciences, Faculty of Environmental and Natural Sciences, University of Southampton, Southampton, United Kingdom; Hosptial Infantil Universitario Niño Jesús, CIBEROBN, SPAIN

## Abstract

Environmental challenges such as a high fat diet during pregnancy can induce changes in offspring growth, metabolism and cardiovascular function. However, challenges that are sustained over several generations can induce progressive compensatory metabolic adjustments in young adults. It is not known if such effects persist during ageing. We investigated whether diets with different fat and carbohydrate contents over three generations modifies markers of ageing. Female C57BL/6 F0 mice were fed diets containing 5% or 21% fat (w/w) throughout pregnancy and lactation. Female offspring were fed the same diet as their dams until the F3 generation. In each generation, body weight, 24-hour food intake were recorded weekly, and plasma metabolites were measured by colorimetric assays, blood pressure by tail cuff plethysmography and vasoconstriction by myography on postnatal day 90 or 456. There was little effect of diet or generation on phenotypic markers in day 90 adults. There was a significant increase in whole body, liver and heart weight with ageing (d456) in the F3 21% fat group compared to the F1 and F3 5% groups. Fasting plasma glucose concentration was significantly increased with ageing in the 5% group in the F3 generation and in the 21% group in both generations. There was a significant effect of diet and generation on ex-vivo vasoconstriction in ageing females. Differences in dietary fat may induce metabolic compensation in young adults that persist over three generations. However, such compensatory effects decline during ageing.

## Introduction

There is evidence that the effects of environmental exposures that induce phenotypic changes can affect more than one generation. For example, in humans nutrition during the pre-pubertal slow growth period has been shown to influence patterns of mortality and health in the grandchildren [1]. Maternal exposure to famine during pregnancy has been shown to induce increased risk of cardio-metabolic disease in the grandchildren [2]. Treatment of pregnant women with diethylstilbesterol (DES) to prevent preterm labour has been associated with increased risk of cancers of the reproductive tract in grandchildren [3,4]. The effects of acute environmental exposures on the phenotype of offspring in subsequent generations have also been reported in rodent models of both nutritional and endocrine challenge. For example, feeding a protein-restricted (PR) diet during pregnancy has been shown to induce altered regulation of glucose homeostasis and impaired vascular function up to the F2 generation [5] and the altered expression of the liver transcriptome up to the F3 generation[6]. Treatment of pregnant rats with dexamethasone induced increased hepatic gluconeogenesis in the F2, but not F3, generation[7]. Furthermore, maternal treatment with the endocrine disruptor vinclozolin induced altered expression of the testis [8] and brain [9] transcriptomes and changes in the DNA methylation of specific genes in the offspring up to the F3 generation.

Induced phenotypic traits do not appear to be passed between generations unchanged and may be progressively lost [7] or modified [3,4,6,10–14]. One possible explanation is that the phenotype induced in each generation reflects the nature of the fetal environment that is influenced by interaction between the physiology of the mother and her environment [15]. For example, exposure of the F0 mother to environmental change can induce physiological change in the F1 offspring and thus the physiological response to the environment during pregnancy in the F1 females may differ from the F0 generation leading to a F2 fetus developing in a different environment to a F1 fetus [15]. Furthermore, because germ cells that will ultimately become gametes and, following fertilisation, the F2 generation offspring are present in the F1 fetus, these cells may be exposed to developmental cues from both F0 and F1 mothers, which represents a potential additional source of phenotypic variation between generations [15].

There have been relatively few experiments that have tested the effect on phenotype of maintaining an environmental challenge over several generations. Increasing total dietary energy intake by 25% in rats over three generations induced impaired regulation of carbohydrate and fat metabolism in the F1 offspring, while fat and glucose homeostasis was improved in F3 offspring despite continued exposure to a higher energy diet [15]. Such metabolic adjustments were accompanied by changes in the epigenetic regulation of specific genes in the liver. These findings are consistent with the suggestion that phenotypic variation between generations can be induced by differences in the interaction between maternal physiology and environment, in this case facilitating progressive metabolic adjustment to the environmental challenge.

Ageing represents a loss of physiological regulation that is influenced by environmental exposures, including nutrition [16]. For example, greater energy intake from dietary fat has been associated with increased physiological decline during ageing including increased weight gain, cognitive decline, loss of musculo-skeletal function and cardiovascular disease [17]. To our knowledge, the effect of dietary patterns maintained over several generations on the ageing phenotype has not been reported.

In the present study we have tested the hypothesis that dietary patterns maintained over three generations alter the phenotype during ageing. To test this, mice were fed diets over three generations containing either 5% or 21% fat, with reciprocal carbohydrate content. We then assessed markers of growth, metabolic control and cardiovascular function in young adult and ageing female animals.

## Methods

### Ethical statement

This study was carried out in accordance with the United Kingdom Home Office Animals (Scientific Procedures) Act (1986) and was conducted under Home Office Licence number 30/2884. The study received institutional approval from the University of Southampton Biomedical Research Facility Research Ethics Committee (no reference number).

### Animals and Diets

The general study design was based upon Hoile *et al* [18). All F0 females were maintained on the standard diet used in the breeding colony (RM1, SDS, Essex, UK) from weaning (Table 1). Virgin female C57BL/6 mice were mated and conception confirmed by presence of a vaginal plug (F0). At conception, F0 females were transferred on to one of two experimental diets containing either 5% or 21% fat by weight derived from corn oil, with a reciprocal decrease in total carbohydrate content (Table 1). The amounts of all other nutrients were the same in the 5% and 21% fat diets. F0 females were allowed to deliver spontaneously at approximately 19 days. Litter size was standardised to a maximum of 8 offspring and biased towards females to ensure sufficient offspring in each study group. Dams were maintained on the same diet during lactation as they were fed during pregnancy. F1 Female offspring were weaned onto the same diet as their mothers on postnatal day 28 and maintained on this diet until postnatal day 90 (young adult) when offspring were divided randomly into three groups. One sub-group was killed by CO_2_ asphyxiation and cervical dislocation. Aortae were removed and placed in physiological salt solution (PSS) for myography. Other organs were collected and snap frozen in liquid nitrogen. Blood was collected via cardiac puncture into tubes containing EDTA, placed on ice and plasma separated from the cells within one hour. All tissues and plasma were stored at -80°C. A second group were mated with stud males that were maintained on RM1 chow diet. Stud males were not mated more than once within a family line. The third group were maintained on the same diet until postnatal day 456 (ageing adult), killed and samples collected as described above. This procedure was repeated for F1 and F2 offspring, but F3 females were not mated. Food intake over 24 hours and body weight were measured at 7-day intervals from postnatal day 35 to day 84, and at one month intervals between day 84 and day 456. Mating success, length of gestation, litter size and litter sex ratio was recorded in each generation. Male offspring were killed at weaning.

**Table 1:** Compositions of the diets.

	Diet		
	2.7%*	5%	21%
Protein (g/kg)	143	160	160
Corn Starch (g/kg)	445	150	150
Maltodextrin (g/kg)	NI	439	279
Sucrose (g/kg)	41	100	100
Total carbohydrate (g/kg)	486	689	529
Corn oil (g/kg)	27	50	210
Cellulose (g/kg)	43	50	50
Choline (g/kg)	1	2.5	2.5
Fiber (g/kg)	177	50	50
Folic acid (mg/kg)	0.8	2.1	2.1
Carbohydrate to fat ratio	19.4	14	2.5
Total metabolisable energy (MJ/kg)	14.7	15.9	19.3

NI, not included in the diet.

*RM1.

### Measurement of metabolites in blood

The concentrations of metabolites in blood were not measured in the same animals at day 90 and at day 456, as it would have been unethical to collect the volume of plasma required to measure the range of metabolites required for the study outcomes twice from the same animal. Therefore, the effect of ageing on the concentrations of metabolites in plasma was assessed by comparison of the subset of females that were allowed to age with the subset of females that were killed as young adults. Plasma glucose (Cayman Chemicals, U.S.A), β-hydroxybutyrate (bHB) (Abcam, U.K), triacylglycerol (TAG) (Cayman Chemicals, U.S.A) and non-esterified fatty acids (NEFA) (Wako, U.S.A) concentrations were measured by colorimetric assays according to the manufacturer’s instructions. Plasma leptin (SPI bio, France) concentration was measured by ELISA according to the manufacturer’s instructions.

### Measurement of blood pressure and heart rate in vivo

Systolic and diastolic blood pressure were measured every 3 months from approximately 84 days of age in each generation by tail-cuff plethysmography using a Columbus Instruments NIBP-8 Non-Invasive Blood Pressure Monitor (Columbus, U.S.A) as described [18]. Briefly, animals were placed in a room heated to 29°C to stimulate tail blood flow. After acclimatisation, mice were placed in a restraining tube and blood pressure cuff placed over the tail and inflated. Five successful blood pressures were recorded, and after the exclusion of the lowest and highest value, the mean of the remaining three measurements was used for data analysis [18].

### Measurement of vasoconstriction ex-vivo

Phenylephrine (Pe)-induced vasoconstriction was measured in aortae by wire myography essentially as described [18]. Briefly, thoracic aortae were placed in PSS (NaCl, 119; KCl, 4.7; CaCl_2_, 2.5; MgSO_4_, 1.17; NaHCO_3_, 25; KH_2_PO_4_, 1.18; EDTA, 0.026; and D-glucose, 5.5 mM) at 4°C. Connective tissue was removed and aorta segments were mounted in a wire myograph (Danish Myo Technology A/S, Denmark), bathed in PSS at 37°C and gassed continuously with 95%(v/v) O_2_ and 5%(v/v) CO_2_. Segments were exposed to 1g tension for 20 minutes before normalisation and functional integrity of segments were assessed by incubation with 125 mM K^+^ in physiological saline solution (KPSS) until tension plateau. Cumulative concentration response curves were then constructed to Pe (1 nM to 100 μM). Vasoconstriction responses were calculated as pEC50 (negative log concentration required to elicit 50% maximal constriction).

### Statistical analysis

Values are expressed as mean ± SEM. Statistical analysis was carried out using SPSS (v22). Group means were compared by ANOVA with generation and dietary fat content as fixed factors, with LSD *post hoc* test. The effect of ageing (difference between day 90 and day 456) was determined using pairwise analysis (un-paired t-test) within each generation and dietary group. Statistical significance was assumed for probabilities <0.05. Based upon a previous study in rats [15], six mice per group was calculated to provide 80% statistical power to detect a 5% difference between groups with a probability of < 0.05 for the outcomes reported in this study.

## Results

Data are presented for F0 and F2 dams corresponding to the F1 and F3 offspring.

### Body weight and energy intake in the dams

Weight gain was significantly lower in the F2 5% dams compared to the F0 5% and F2 21% dams (interactive effect of diet*generation (F(1,26) = 10.55, P = 0.03) (S1 Fig).

There was a significant effect of diet (F(1,23) = 19.26, P < 0.0001), and a significant interactive effect of generation*diet (F(1,23) = 11.60, P = 0.002) on energy intake over the first 14 days of pregnancy (S1 Fig). In the F0 generation, energy intake over the 14 days of pregnancy, summarised as area-under-the-curve (AUC), corrected for body weight, was significantly increased in the 21% group, compared to the 5% group. In the F2 generation energy intake was significantly increased in the 21% dams, compared to the 5% dams. Across both the 5% and 21% lineages, energy intake during the first 14 days of pregnancy was significantly decreased in the F2 generation, compared to the F1 generation.

### Assessment of reproductive efficiency

There were no statistically significant differences between dietary groups or generations in the length of gestation, the size of the litter, the weight of the litter or in the ratio of males to females (Table 2).

**Table 2:** Mating success and pregnancy outcomes.

Dietary group (% fat)				
	5%		21%	
Generation	F0	F2	F0	F2
Successful pregnancy (%)^1^ ^,^ ^2^	77	73	69	88
Dam ate offspring (%)^1^	0	27	15	0
Gestation (days)	19.3±0.3	20.0±0.7	18.6±0.2	18.1±0.7
Litter size	7.6±0.5	5.9±0.2	7.2±0.7	7.7±0.4
Litter weight (g)	11.4±0.5	11.1±1.0	10.1±0.9	8.5±1.1
M:F ratio	1.2±0.2	1.3±0.5	1.5±0.3	1.2±0.2

^1^Values are expressed as a proportion of mating’s. All other values are expressed as mean ± SEM.

^2^Successful pregnancy was defined as spontaneous delivery of the litter without subsequent cannibalism of the offspring by the dam. Means with different superscripts differed significantly.

### Assessment of energy intake and growth in young adults

**Fig 1:**
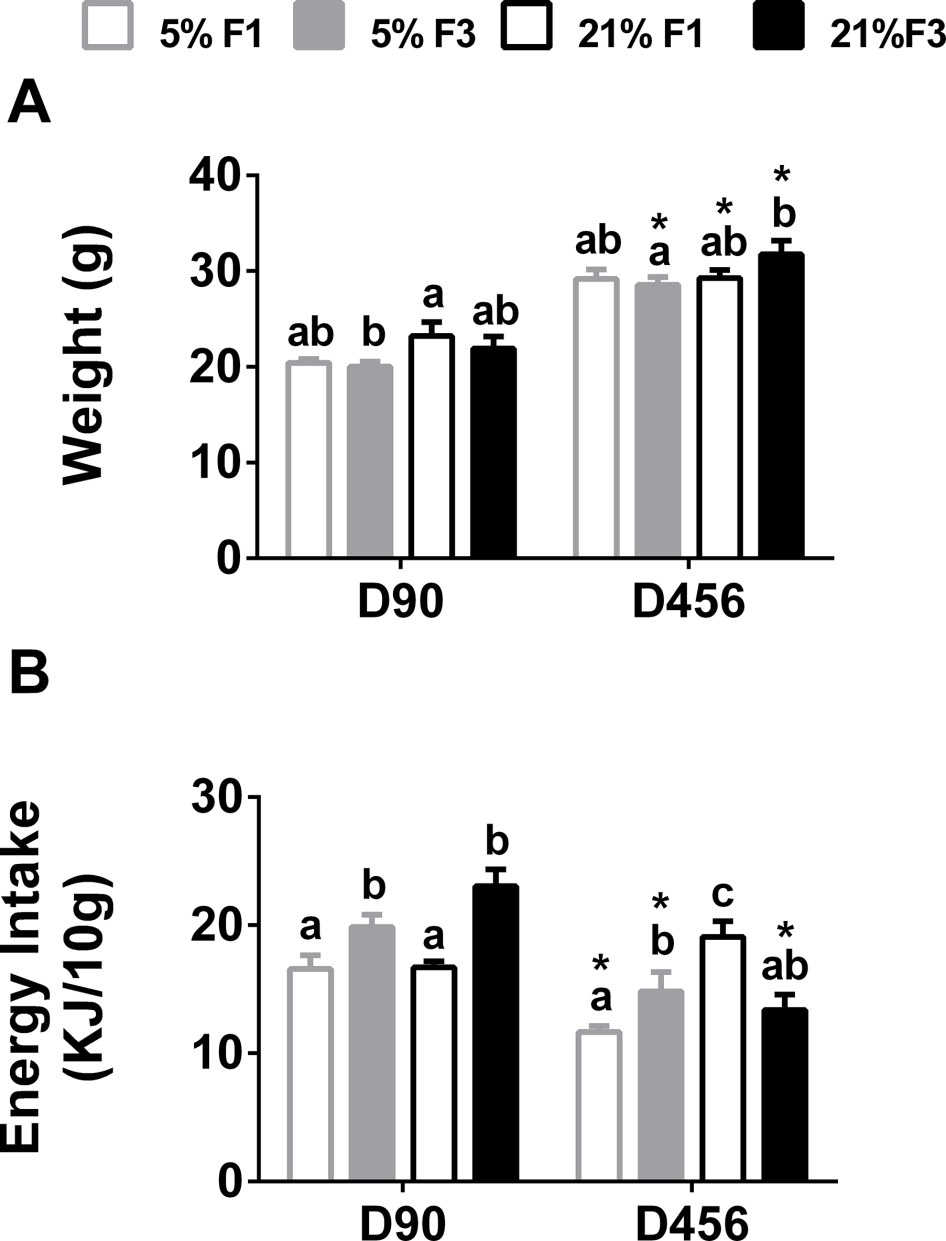
(A) Body weight and (B) energy intake in young (D90) and ageing (D456) adult mice. Values are mean ± SEM (n = 5 to 7 per dietary group per generation). Statistical comparison between groups, within each age, was carried out by ANOVA with diet and generation as fixed factors, with LSD *post hoc* correction. Means with different letters differ significantly (P < 0.05). *Means significantly different (P<0.05) between D456 and D90, determined by Student’s unpaired t-test (P<0.05).

There was a significant effect of diet on body weight in young adults (F(1,23) = 5.686, P = 0.026) (Fig 1A). There was no difference in body weight between dietary groups in either the F1 or F3 generation. There were no significant differences across either the 5% or 21% dietary lineages. Body weight was decreased significantly in the F3 5% group, compared to the F1 21% group.

There was a significant effect of generation on 24-hour energy intake across in young adult mice (F(1,23) = 10.916, P = 0.003) (Fig 1B). There were no differences between dietary groups on either the F1 or F3 generation. In both the 5% and 21% dietary lineage 24-hour energy intake at day 90 was increased significantly in the F3 generation, compared to the F1 generation.

**Fig 2:**
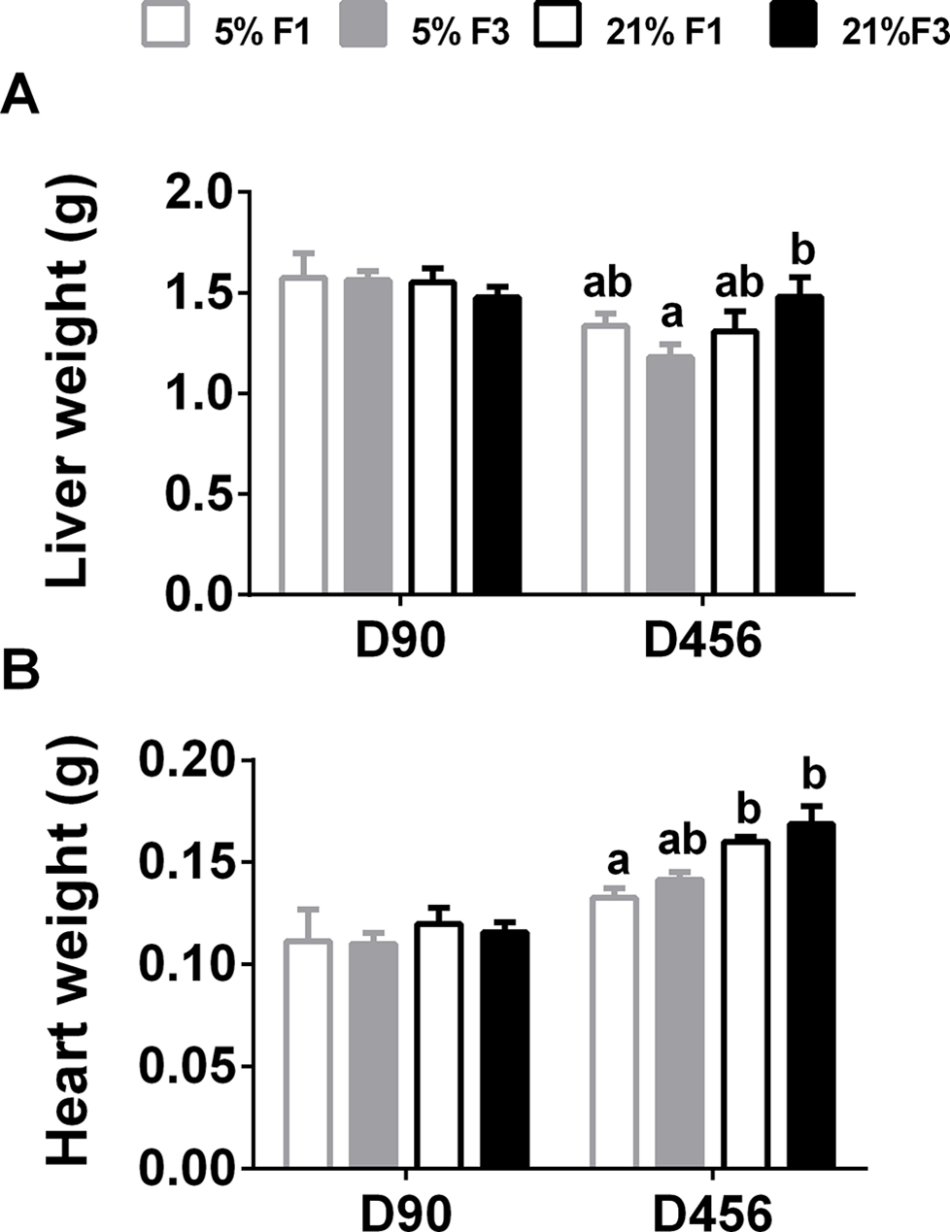
(A) Liver and (B) heart weight in young (D90) and ageing (D456) adult mice. Values are mean ± SEM (n = 5 to 7 per dietary group per generation). Statistical comparison between groups, within each age, was carried out by ANOVA with diet and generation as fixed factors, with LSD *post hoc* correction. Means with different letters differ significantly (P < 0.05). *Means significantly different (P<0.05) between D456 and D90, determined by Student’s unpaired t-test (P<0.05).

There were no significant differences in liver or heart weight in young adults (Fig 2).

### Assessment of plasma metabolites in young adults

**Fig 3:**
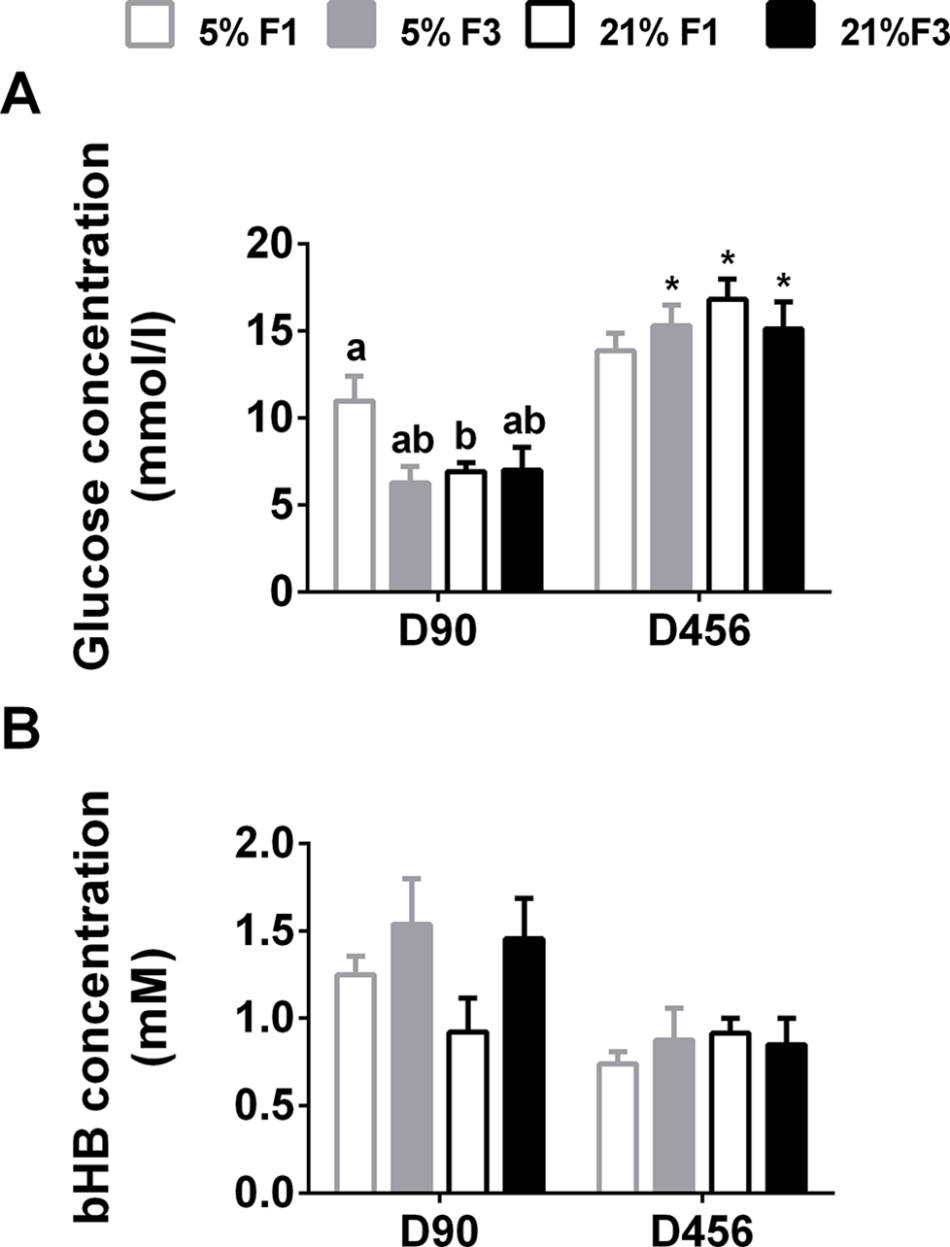
Fasting (A) glucose and (B) bHB plasma concentrations in young (D90) and ageing (D456) adult mice. Values are mean ± SEM (n = 5 to 7 per dietary group per generation). Statistical comparison between groups, within each age, was carried out by ANOVA with diet and generation as fixed factors, with LSD *post hoc* correction. Means with different letters differ significantly (P < 0.05). *Means significantly different (P<0.05) between D456 and D90, determined by Student’s unpaired t-test (P<0.05).

There was a significant interactive effect of diet*generation on plasma glucose concentration in offspring at day 90 (F(1,23) = 4.411, P = 0.047) (Fig 3A). In the F1 generation, plasma glucose was significantly lower in the 21% group compared to the 5% group, in the F1 generation. There was no difference between dietary groups in the F3 generation or across dietary lineages. There were no significant effects of diet or generation on fasting plasma bHB concentration in young adults (Fig 3B).

### Assessment of cardiovascular outcomes and, blood vessel sensitivity in young adults

**Fig 4:**
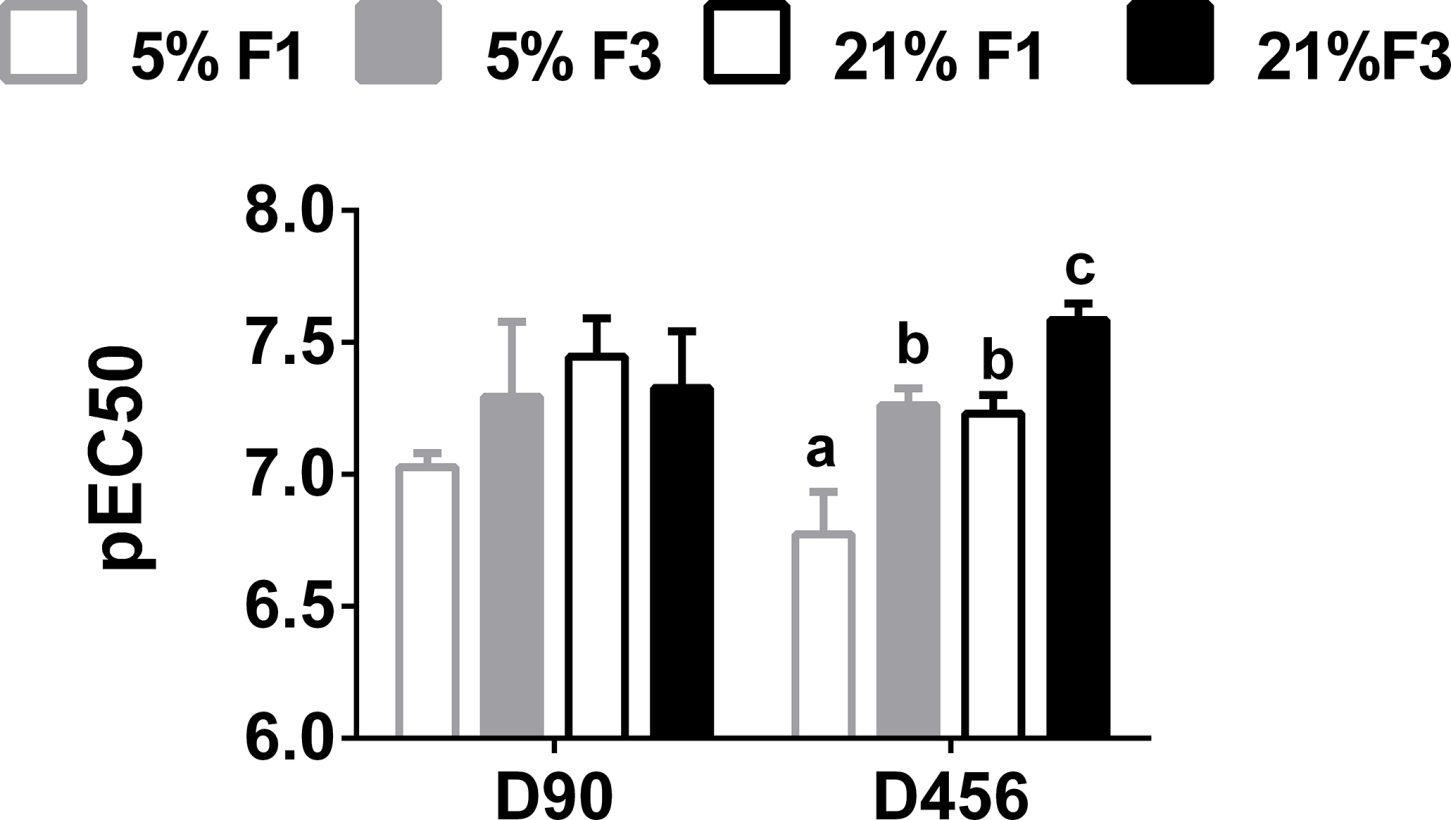
Pe induced vasoconstriction (pEC50) in young (D90) and ageing (D456) adult mice. Values are mean ± SEM (n = 5 to 7 per dietary group per generation). Statistical comparison between groups, within each age, was carried out by ANOVA with diet and generation as fixed factors, with LSD *post hoc* correction. Means with different letters differ significantly (P < 0.05). *Means significantly different (P<0.05) between D456 and D90, determined by Student’s unpaired t-test (P<0.05).

There were no significant effects of diet or generation on BP or vasoconstriction (S2 Fig). There were no significant effect of diet or generation on vasoconstriction response to Pe *ex-vivo* (Fig 4).

### Assessment of energy intake and growth in ageing mice

The effect of ageing on phenotypic outcomes was assessed in a subset of females. There were no significant overall effects of diet or generation on body weight in D456 between young and ageing animals. In the F3 generation, body weight was significantly higher in the 21% females, compared to the 5% females. Body weight was significantly higher in D456 females, compared to corresponding D90 females in the F1 21% and F3 5% and 21% groups (Fig 1A). There was a significant effect of diet (F(1,26) = 6.784, P = 0.015) and a significant interactive effect of diet*generation diet (F(1,26) = 15.118, P = 0.001) on energy intake in ageing females (Fig 1B). Energy intake was significantly higher in the F1 21% and F3 5% groups, compared to the F1 5% group and significantly lower in the F3 21% group, compared to the F1 21% group. Energy intake at D456 was significantly lower than at D90 for F1 5%, F3 5% and F3 21% groups.

There was a significant interactive effect of diet*generation (F(1,31) = 4.545, P = 0.041) on liver weight (Fig 2A). There was no significant difference in liver weight in the F1 generation, however in the F3 generation liver weight was significantly increased in the 21% females. There was a significant effect of diet on heart weight (F(1,31) = 23.480, P < 0.001) (Fig 2B). Heart weight was significantly higher in the F1 21% group, compared to the F1 5% group, this difference was lost by the F3 generation (Fig 2B). There were no differences in organ weight between D90 and D456 females.

### Comparison of the concentrations of metabolites in the plasma of ageing mice

The effect of ageing on metabolic outcomes was assessed in a subset of females. There was no significant effects of diet or generation on fasting plasma glucose concentration in D456 females (Fig 3A). Fasting plasma glucose concentration was significantly higher in D456 females in the F3 5%, F1 21% and F3 21% groups, compared to their D90 counterparts.

There was no significant effects of diet or generation on fasting plasma bHB concentration in D456 females (Fig 3B). There were no differences in fasting plasma bHB concentration between D90 and D456 females.

### Comparison of cardiovascular outcomes in ageing adult mice

Systolic and diastolic blood pressures were measured in the same animals on day 90 and on day 456. There were no significant effects of age, generation or diet on SBP or DBP (S2 Fig).

There was a significant effect of diet (F(1,18) = 13.944 P = 0.002) and generation (F(1,18) = 16.297 P = 0.001) on vasoconstriction response to Pe *ex-vivo*. pEC50 was significantly increased in the F3 generation, compared to the F1 generation, within each dietary lineage, where pEC50 for the 21% groups was significantly higher than that of the 5% groups in each generation (Fig 4).

## Discussion

The findings of the present study show that while young mice were able to compensate exposure for differences in fat and carbohydrate intake over three generations, this effect was impaired in aged mice in a manner contingent on the composition of their diet.

Previous data has shown that exposure to dietary challenge over several generations can induce compensatory metabolic changes in successive generations that facilitate homeostasis in young adult rodents [15]. Such compensatory processes appear to involve developmental plasticity acting via epigenetic mechanisms that may reflect progressive changes in the developmental environment arising from differences between generations in the interaction between physiology of the pregnant mother and her environment [15]. The present findings are consistent with this view in that changing the diet of the F0 dams at conception to a diet containing 5% or 21% fat, which was then sustained over subsequent generations, was associated with a decrease in maternal energy intake during pregnancy between the F0 and F2 generations. Such compensatory changes in energy intake have been reported in several species [19–24] and appear to involve sensing of both the fat and carbohydrate content of the diet [25]. However, in contrast to previous studies, the apparent adjustments in food intake occurred over several generations rather than in one generation. One implication of this finding is that appetite can be regulated over more than one generation, although the underlying mechanism cannot be deduced form the present data. However, both under- and over- nutrition in early life have been shown previously to induce changes in the epigenetic regulation of key appetite genes, for example proopiomelanocortin [26,27]. This suggests that changes in epigenetic processes may be a mechanism by which patterns of food intake could be modified across generations.

In day 90 females, dietary fat content altered 24-hour energy intake and fasting plasma glucose levels only. Energy intake was increased significantly in the F3 generation, compared to the F1 generation, irrespective of diet. Interestingly, body weight was unaltered, suggesting the energy requirement to reach the specific body weight by D90 has changed across generations. This implies a compensatory metabolic response to the increased energy content of the diet, which is specific to each generation. Fasting plasma glucose was significantly lower in the 21% group compared to the 5% group in the F1 generation, while there were no significant differences between dietary groups in the F3 generation due to reduction in plasma glucose concentration in the 5% group. One possible interpretation is that both dietary groups underwent metabolic adjustment to increased fat intake, but the rate for changes was faster in the 21% group than the 5% group. This may reflect the lower carbohydrate content of the 21% fat compared to the 5% diet, or that the greater dietary change induced a more marked metabolic response. Thus despite the difference in dietary fat exposure and the duration of the nutritional differences, there were no significant effects on any other measured outcome, including blood pressure which has been shown previously to be modified by dietary fat in in rodents [28–32]. Thus there was no indication of a cumulative effect of diet between generations on the phenotype of the young adult mice, which suggests induction of physiological compensations that operate across generations.

Ageing was associated with diet and generation related changes in some phenotypic outcomes that were not present in young adults. The F3 21% group display an increase in liver and heart weight with ageing, in contrast to the other groups. Since dietary fat fed during pregnancy or to free living offspring has been shown to increase liver fat content [33], one possible explanation is increased fat deposition in these tissues. Fasting glucose concentrations were significantly higher in ageing mice in the F3 5% and both the F1 and F3 21% groups, compared to their D90 counterparts and the F1 5% group. This may reflect lower insulin levels, decreased insulin sensitivity or increased hepatic gluconeogenesis [34]. Because plasma availability was limited it was not possible to measure insulin or glucagon concentrations in this study. In contrast, energy intake or body weight were not altered significantly by ageing.

Ageing has been associated causally with decreased arterial elasticity that is associated with increased vasoconstriction [35]. Feeding high fat diets during pregnancy or adulthood in rats has been shown to increase vasoconstriction and blood pressure [29,36–40], although the precise effect may be related to the type of dietary fat [36,40].

The results here suggest a significant cumulative effect of diet between generations on vasoconstriction in the ageing mice. One possible interpretation is that any compensatory effects to dietary content are at the expense of vascular function during ageing. A similar consequence of compensation was observed in our previous transgenerational study, whereby adult female rats regained fasting metabolic control over the course of three generations whilst displaying an increase in plasma corticosterone levels [15].

**Fig 5:**
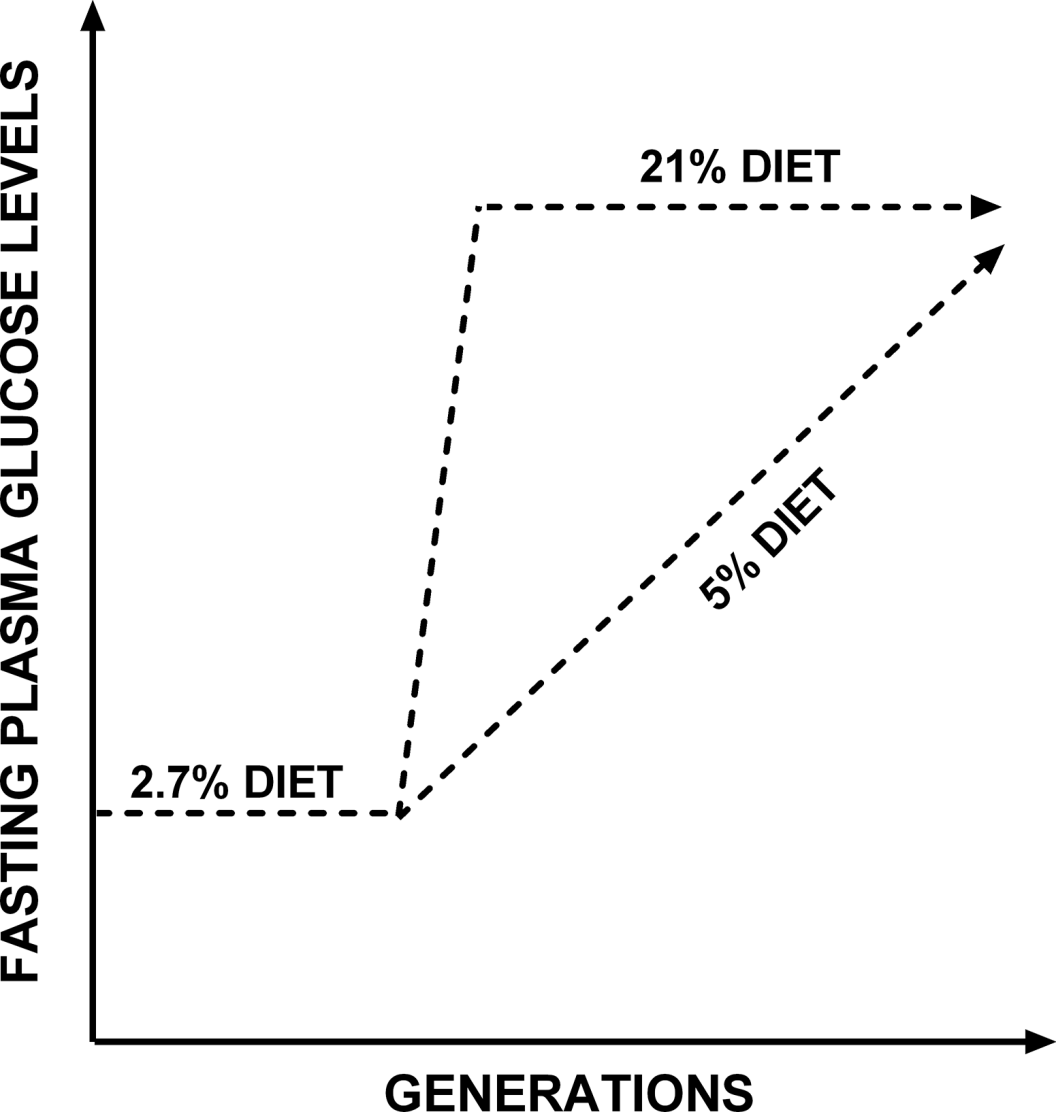
Schematic illustrating the effect of multiple generation exposure to increased dietary fat and carbohydrate on changes in fasting plasma glucose levels with ageing. The effect of the 21% is evident form the initial generation exposed to the diet and remains unchanged following multiple generations on the diet. Exposure to the 5% diet leads to few or no effects in the initial generation exposed, however following a sustained exposure to the diet for three generations, a similar profile is reached to that of the 21% exposed group.

These findings suggest that dietary transition followed by sustained exposure to an altered diet over three generations can induce compensatory physiological adjustments in young adult mice. However, some, but not all, of these apparent adjustments are lost during ageing in a manner that is related to the amount of fat in the diet. Deterioration in specific physiological outcomes occurs over fewer generations in animals fed the higher fat diet (Fig 5). Whether the trajectory of phenotypic change in ageing is related to the magnitude of the initial dietary transition during pregnancy in the F0 generation or to the composition of the post-transitional diet cannot be deduced from the current data. In females, particularly in rodents, regulation of energy balance is associated closely with reproductive capacity, where deficits in the quality or abundance of nutrition may have negative effects on pregnancy [41,42]. If a drive to maintain energy homeostasis in order to achieve optimal reproductive outcome were to underlie the mechanisms that facilitate compensation against variations in energy intake, then one explanation for lack of adjustment in some physiological outcomes, such as glucose homeostasis, may be a biological trade-off for maintaining reproductive function.

## Supporting Information

S1 Fig (A) Body weight (AUC, area-under-the-curve) and (B) energy intake (AUC) in pregnant dams. Values are mean ± SEM (n = 9 to 11 per dietary group per generation). Statistical analysis was carried out by ANOVA with diet and generation as fixed factors, with LSD post hoc correction. Means with different letters differ significantly (P < 0.05).
(TIF)

S2 Fig (A) Systolic and (B) diastolic blood pressure in young (D90) and ageing (D456) adult mice. Values are mean ± SEM (n = 5 to 7 per dietary group per generation). Statistical comparison between groups, within each age, was carried out by ANOVA with diet and generation as fixed factors, with LSD *post hoc* correction. Means with different letters differ significantly (P < 0.05). *Means significantly different (P<0.05) between D456 and D90, determined by Student’s unpaired t-test (P<0.05).
(TIF)
