# Correction: Fat and Carbohydrate Intake over Three Generations Modify Growth, Metabolism and Cardiovascular Phenotype in Female Mice in an Age-Related Manner

**DOI:** 10.1371/journal.pone.0189655

**Published:** 2017-12-07

**Authors:** Samuel P. Hoile, Leonie R. Grenfell, Mark A. Hanson, Karen A. Lillycrop, Graham C. Burdge

The second author's middle initial is incorrect. The correct name is: Leonie R. Grenfell. The correct citation is: Hoile SP, Grenfell LR, Hanson MA, Lillycrop KA, Burdge GC (2015) Fat and Carbohydrate Intake over Three Generations Modify Growth, Metabolism and Cardiovascular Phenotype in Female Mice in an Age-Related Manner. PLoS ONE10(8): e0134664. https://doi.org/10.1371/journal.pone.0134664

## References

[1] Hoile SP, Grenfell LM, Hanson MA, Lillycrop KA, Burdge GC (2015) Fat and Carbohydrate Intake over Three Generations Modify Growth, Metabolism and Cardiovascular Phenotype in Female Mice in an Age-Related Manner. PLoS ONE 10(8): e0134664 https://doi.org/10.1371/journal.pone.0134664 26266533 10.1371/journal.pone.0134664 PMC4534415

